# Systematic Review and Meta-Analysis to Establish the Association of Common Genetic Variations in Vitamin D Binding Protein With Chronic Obstructive Pulmonary Disease

**DOI:** 10.3389/fgene.2019.00413

**Published:** 2019-05-16

**Authors:** Ritesh Khanna, Debparna Nandy, Sabyasachi Senapati

**Affiliations:** Department of Human Genetics and Molecular Medicine, Central University of Punjab, Bathinda, India

**Keywords:** vitamin D-binding protein, COPD, meta-analysis, linkage disequilibrium, genetic polymorphisms, allelic heterogeneity

## Abstract

**Background:** Vitamin-D binding protein (DBP) also known as GC protein, is a major determinant for vitamin- D metabolism and transport. GC1F, GC1S, and GC2 are the three allelic variants (denoted as rs4588 and rs7041) of GC, and known to be associated with chronic obstructive pulmonary disease (COPD). However, contradictory reports and population specific risk attributed by these alleles warranted detailed genetic epidemiology study to establish the association between GC variants and COPD. In this study we performed a meta-analysis and investigated the genetic architecture of GC locus to establish the association and uncover the plausible reason for allelic heterogeneity.

**Methods:** Published cross-sectional case control studies were screened and meta-analysis was performed between GC variants and COPD outcome. RevMan-v5.3 software was used to perform random and/or fixed models to calculate pooled odds ratio (Meta-OR). Linkage disequilibrium (LD) and haplotypes at GC locus were evaluated using 1000 Genomes genotype data. *In silico* functional implications of rs4588 and rs7041 was tested using publicly available tools.

**Results:** GC1F allele and GC1F/1F genotype were found to confer COPD risk in overall meta-analysis. GC1S/1S was found to confer risk only among Europeans. *In silico* investigation of rs4588 and rs7041 identified strong eQTL effects and potential role in regulation of GC expression. Large differences in allele frequencies, linkage disequilibrium (LD) and haplotypes were identified at GC locus across different populations (Japanese, African, Europeans, and Indians), which may explain the variable association of different GC alleles in different populations.

**Conclusion:** GC1F and GC1F/1F impose significant genetic risk for COPD, among Asians. Considerable differences in allele frequencies and LD structure in GC locus may impose population specific risk.

## Introduction

Chronic obstructive pulmonary disease (COPD) is a complex disease affecting the lung function. Genetically susceptible individuals develop the COPD while they get exposed to environmental triggers, such as noxious gases or suspended particles. Decreased level of vitamin-D in serum is associated with COPD among individuals with a history of smoking (Janssens et al., 2010). Besides environmental and genetic factors, metabolic factors are also critical and do cross talk with each other for the pathogenesis of COPD (Rabe et al., 2007).

Vitamin-D binding protein (DBP), also known as group-specific component (GC), belongs to a gene cluster family which is expressed in liver and other tissues (Chishimba et al., 2010). As the name suggests, it is known for its binding to circulating vitamin-D_3_ and its transportation from liver to other tissues during its metabolism (Daiger et al., 1975). *GC* is a highly polymorphic gene and three of its allelic variants, namely *GC*1F, *GC*1S, and *GC*2, have been studied extensively for their association with vitamin-D deficiency (VDD) and other diseases including COPD (Chishimba et al., 2010; Wood et al., 2011). These variants correspond to different allelic arrangements of rs7041 and rs4588 (Table 1). These GC protein variants are reported to have a different affinity to bind to vitamin-D_3_ i.e., 25(OH)D_3_, and thus affect its serum concentration (Arnaud and Constans, 1993; Janssens et al., 2010). Circulating level of vitamin-D_3_ is regulated by the synthesis and enzymatic degradation of 25(OH)D_3_ by catabolizing enzymes. More than 90% of the circulating 25(OH)D_3_ present in tightly bound (K_d_ ~ 10^−9^ M) form with GC proteins (Arnaud and Constans, 1993). Therefore, different GC isoforms influence the serum concentration/bioavailability of 25(OH)D_3_.

**Table 1:** Allelic arrangements correspond to three different GC variants implicated in COPD.

**GC variants**	**Allele of rs4588 (amino acid)**	**Allele of rs7041(amino acid)**
GC-1F	C (Thr436)	T (Asp432)
GC-1S	C (Thr436)	A/G (Glu432)
GC-2	A (Lys436)	T (Asp432)

A study performed on north Indian cohort has shown homozygous *GC*1F variant to confer risk, and the disease severity is observed in a variant specific dose dependent manner, where the geometric mean of serum 25(OH)D_3_ was observed in the ascending order of *GC* genotypes 1F/1F <1S/1F <1S/1S <1S/2 <2/2 among COPD patients (Maheswari et al., 2014). Recent reports also indicate the protective role of *GC*2 variant among healthy individuals. Similar reports were published by the studies done among Caucasian and north Indian cohorts (Schellenberg et al., 1998; Berg et al., 2013; Maheswari et al., 2014; Chen et al., 2015). Azzawi et al. confirmed similar study outcomes in an Egyptian cohort where *GC*1F and *GC*1F/1S variants were found to be associated with low serum vitamin-D_3_ concentration (Al-Azzawi et al., 2017). While studies done among Korean population have shown an association of *GC*2 variants with COPD progression, where *GC*2 and *GC*1F/1S variants were shown to be associated with higher emphysema index, irrespective of VDD. These studies also identified an association of *GC*2 and *GC*1F/1S variants with lower and higher serum concentration of vitamin-D_3_, respectively. *GC2* showed significant association with VDD (Jung et al., 2014; Park et al., 2016).

GC protein (or DBP) is also involved in the inflammation by getting converted into MAF (Macrophage Activating Factor) in the presence of enzymes secreted by leucocytes. It has been found that the conversion of GC into GC-MAF is a deglycosylation process. Absence of glycosylated *Lys* residue at 420 in *GC*2 variants makes it an inappropriate reactant for the deglycosylation process, which makes them protective for COPD (Maheswari et al., 2014). Vitamin-D_3_ is known to inhibit the expression of MMPs (Matrix Metalloproteinases), which are responsible for the emphysema degradation of lung alveoli. Thus, optimal serum concentration of Vitamin-D_3_ is very critical among emphysema patients and a trial for such serum Vitamin D_3_ intervention among a large participant group can further elucidate its role in COPD progression (Berg et al., 2013). Serum Vitamin-D_3_ is also found to have seasonal and geographical variations, which depend on the amount of sunlight reaching the skin (Jung et al., 2014; Al-Azzawi et al., 2017). ECLIPSE Cohort study did not find an association between serum DBP and emphysema or lung function, although a negative correlation was found among DBP and serum 25(OH)D_3_ level (Berg et al., 2013). While another study in an alpha1-antitrypsin deficient Caucasian population showed the association of serum DBP with COPD conditions (Wood et al., 2011). A recent report indicated a strong relationship between serum 25(OH)D_3_ and pulmonary function (FEV1 and FVC) in a well-defined COPD cohort (Janssens et al., 2010).

It is evident that GC is a major determinant for several health parameters including those associated with COPD. However, contradictory findings of association of different alleles with COPD and non-replication across different populations warranted further meta-analysis and detailed population genetics studies. In the present study, we anticipated to explain the association of known *GC* alleles with COPD and investigate the genetic and functional aspects of *GC* alleles. Locus architecture of different populations was also investigated to explain the non-replication/differential replication of *GC* alleles in different populations. We hypothesized that genetics architecture at *GC* locus leads to population specific allelic variation in *GC* and its association with COPD. The study was performed with the following specific objectives: (i) perform meta-analysis to establish association of commonly studied *GC* alleles with COPD, and (ii) investigate the genetic heterogeneity at a functionally relevant *GC* locus, that explain variability in GC protein and COPD.

## Materials and Methods

### Literature Retrieval

Our objective was to identify research articles where genetic association of *GC* has been tested with COPD. We restricted our study to three major genetic polymorphisms of *GC*, namely *GC*1F, *GC*1S, and *GC*2 alleles. Literature was searched online in the National Center for Biotechnology Information (NCBI-PubMed), Google Scholar and Medline. The major search language for the literature was English, papers in other languages were translated for further review. To obtain the best quality outcome, we include only peer reviewed scientific literature. Literature were searched until May 2018. The keywords used for the search for literature were as follows: Vitamin D binding protein and chronic obstructive pulmonary disease, DBP and COPD, *GC* alleles and COPD, COPD association *GC*. Cross references were also reviewed and references from the retrieved articles were also checked manually so as to find any relevant articles.

### Inclusion and Exclusion Criteria

Only case-control studies were included for this meta-analysis. Only those studies were included where different alleles (1F, 1S, and 2) and genotypes (1F/1F, 1S/1S, 2/2, 1F/1S, 1F/2, 1S/2) of *GC* were studied for their association with COPD. Included studies clearly mentioned either the actual numbers, or the percentage of cases and controls with different genotypes and alleles of *GC*. Included studies have both smokers and non-smokers among both cases and controls.

### Data Extraction

Data was extracted from eligible articles by two investigators independently and differences and controversies were resolved by group discussions. We first validated the study types and then extracted author names, year of publication, details of genotypes/alleles and their frequencies in COPD patients and controls.

### Statistical Analysis

Results of association of three distinct alleles have been included in this study. These alleles were *GC*1F, *GC*1S and *GC*2, represented in NCBI dbSNP as rs4588 and rs7041, respectively (Table 1). Therefore, a total of six different genotypic combinations were studied, such as, *GC*1F/1F, *GC*1F/1S, *GC*1S/1S, *GC*1F/2, *GC*1S/2, and *GC*2/2. Independently these genotypes and three allelic associations were evaluated by meta-analysis. In each analysis, the experimental allele or genotypes were tested against the total allele or genotype counts. Meta-analysis was performed using Review Manager (RevMan-v5.3) Copenhagen: The Nordic Cochrane Center, The Cochrane Collaboration, 2014. Additive genetic model with 95% confidence interval (CI) was used in each of these independent analyses. Heterogeneity between studies was calculated by the *I*^2^ and chi^2^ test, where *I*^2^> 50% and chi^2^ *p* < 0.05 was considered as significant heterogeneity. Meta-analysis of odds ratios were performed using a random effect model where significant heterogeneity was observed, otherwise a fixed effect model was used. Overall effect size (Meta-OR) was calculated by Z-test with 5% alpha level. A sensitivity analysis was performed to access whether meta-analysis results were substantially influenced by the presence of any study. This was done by systematically excluding one study at a time and recalculating the significance (*p*-value of the χ^2^ and *Z*-test) of the results. The funnel plot was used to analyze the publication bias. Subgroup analysis between Asian and Caucasian studies was also performed to identify any significant differences due to individual group stratification.

### Linkage Disequilibrium, Haplotypes, and Comparative Allele Frequency

Genetic architecture of *GC* locus was evaluated to explain population specific effects (if any) of *GC* alleles on its association with different human traits/diseases. To analyze the linkage disequilibrium, LD plots and haplotypes were reconstructed using Haploview (Barrett et al., 2004). LD calculations and manipulation of genotype files were done using Plink 1.07 (Purcell et al., 2007). 1000 Genomes genotype information for four major populations, such as CEU (Utah residents with northern and western European ancestry), GIH (Gujarati Indians in Houston, USA), YRI (Yoruba in Ibadan, Nigeria), and JPT (Japanese in Tokyo), were evaluated for LD analysis. Raw genotype data for these populations were obtained from 1,000 Genomes ftp through Ensembl. Genotype data were obtained for a 50 kb window on both the sides around rs7041 i.e., chr4:71702617-71802617 (GRCh38.p12). Comparative allele frequencies for *GC*1F, *GC*1S, and *GC*2 corresponding to rs4588 and rs7041 were evaluated from Ensembl (https://asia.ensembl.org/index.html), HaploReg (http://archive.broadinstitute.org/mammals/haploreg/haploreg.php).

### *In silico* Functional Implication Assessment

Functional implications of rs4588 and rs7041 were analyzed using open source browsers. RegulomeDB (http://www.regulomedb.org/index) was used to analyze the regulatory function and GTEx portal (https://gtexportal.org/home/) was used to analyze single tissue or gene eQTL.

## Results

### Characteristics of Eligible Studies

**Figure 1:**
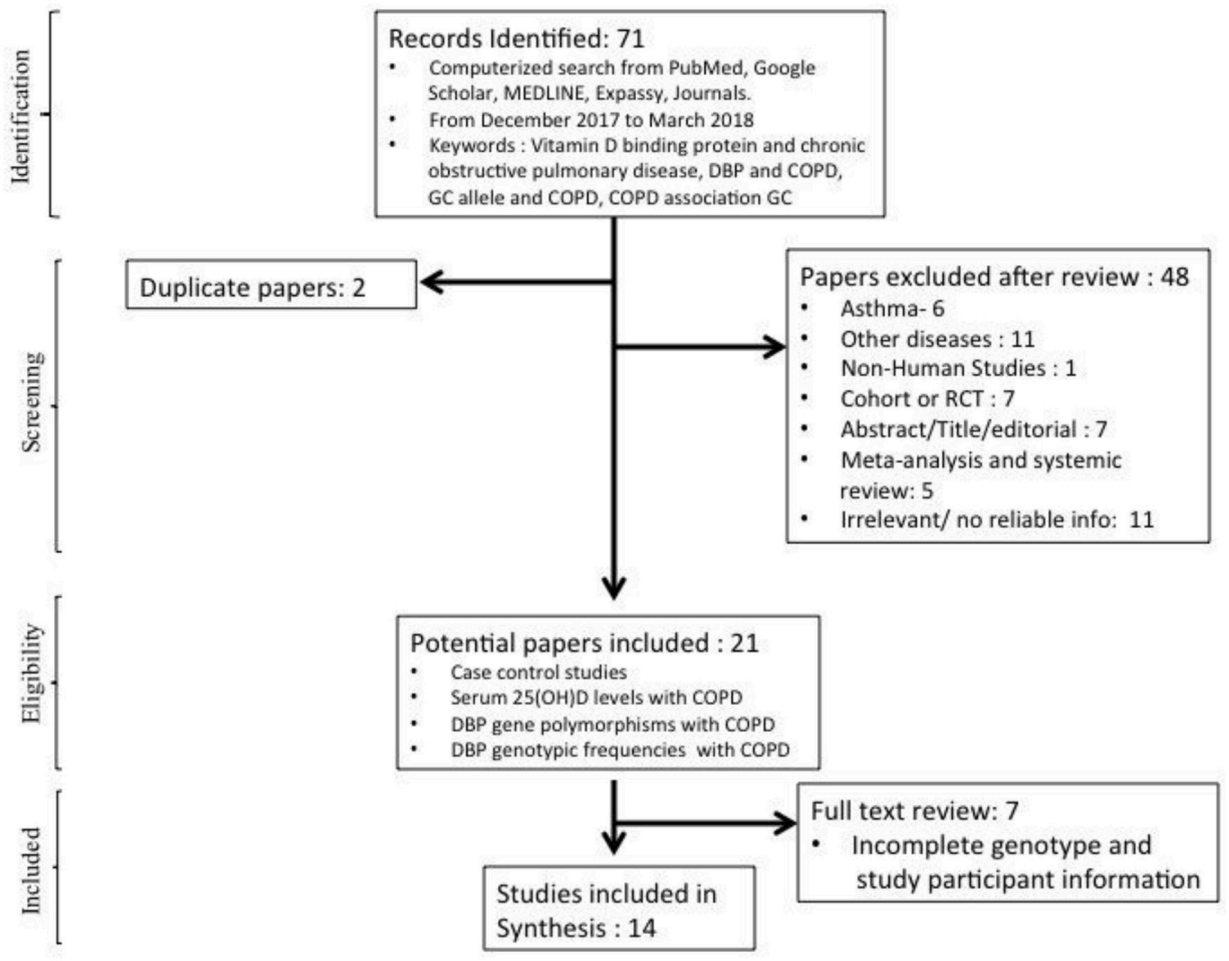
Step-wise flow chart showing identification and screening of studies included in the meta-analysis.

A total of 71 studies were identified initially after online literature search. After screening and proper reviewing for the eligible papers 48 papers were excluded. There were two duplicate studies, six studies were for asthma, and 11 were for diseases other than asthma and COPD, such as osteomalacia, type II diabetes, adenocarcinoma, pulmonary tuberculosis and other non-relevant diseases. There was a non-human study done on mice, which was also excluded from the meta-analysis. Meta-analysis (*n* = 5), which was done previously on COPD and *GC*, was also excluded but was used to identify cross references. Fourteen studies were excluded because they were either cohort studies or random clinical trials done on supplementation of vitamin-D_3_. Eleven studies were found irrelevant, either due to less information for cases or control subjects, and one study was in other language and was excluded from the meta-analysis. A further seven studies were excluded as adequate/complete genotype and study participant information were not given. After this screening based on our inclusion/exclusion criteria, a total of 14 studies were found eligible for meta-analysis (Kueppers et al., 1977; Home et al., 1990; Ishii et al., 2001; Ito et al., 2004; Laufs et al., 2004; Lu et al., 2004; Korytina et al., 2006; Huang et al., 2007; Janssens et al., 2010; Shen et al., 2010; Jung et al., 2014; Li et al., 2014; Maheswari et al., 2014; Al-Azzawi et al., 2017) (Figure 1).

### Genotypic and Allelic Association

**Figure 2:**
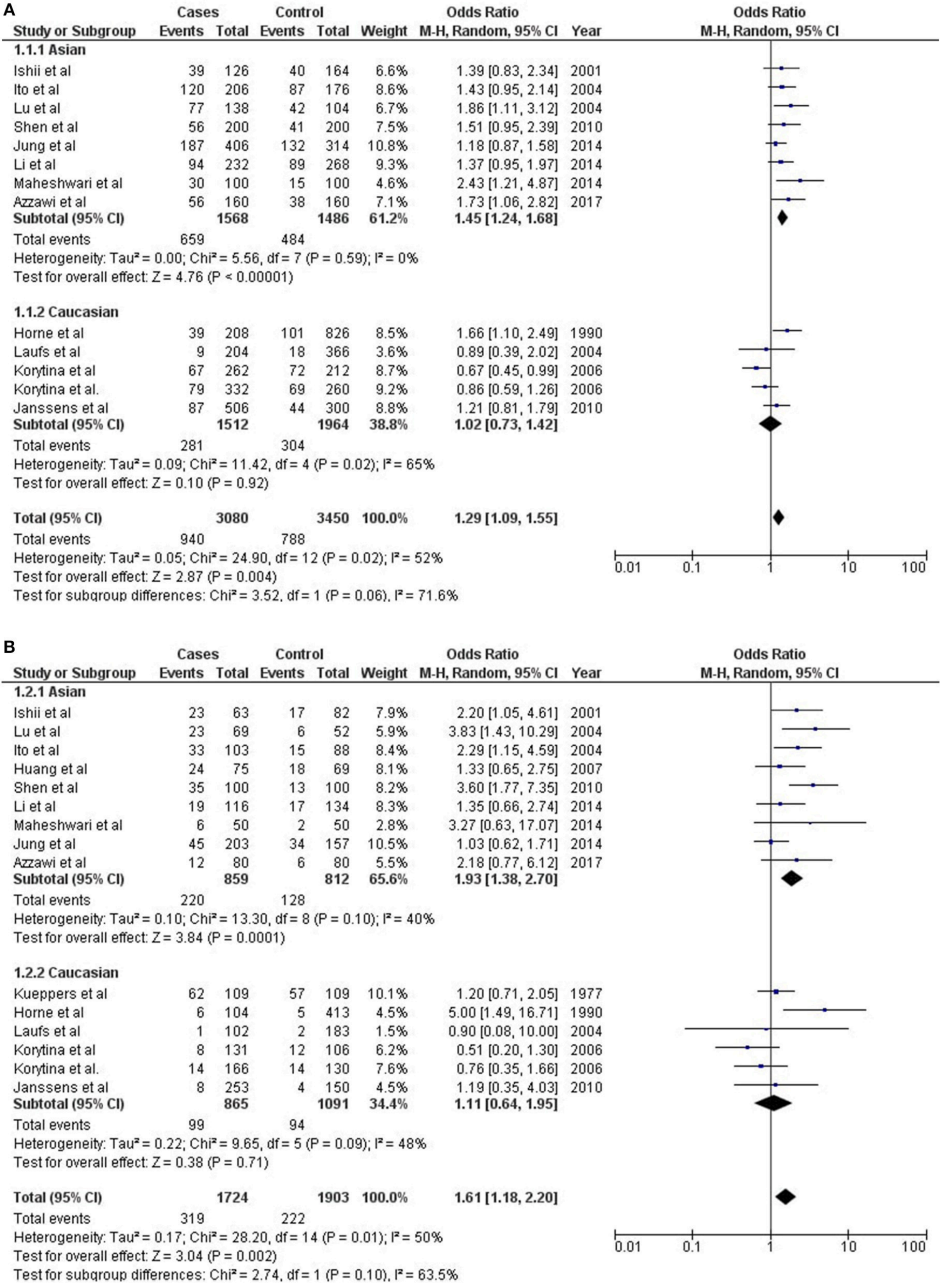
Assessment of risk for meta-analysis of **(A)** *GC1F* allele, and **(B)** GC1F/1F genotype with COPD.

A total of 14 studies were included in this meta-analysis where genotypes for different above-mentioned *GC* alleles, in both COPD patients and healthy controls, were reported. Out of these 14 studies, nine studies were performed on different Asian populations and five were on European populations. Details of the study participants and haplotypes or allele frequencies are given in the Supplementary Table 1. Random effect model was performed to find out the pooled effect size for *GC*1F/1F, *GC*1F/S, *GC*1F/2, and *GC*2/2 genotypes, and *GC*1F, *GC*1S and *GC*2 alleles in COPD. For remaining analyses, fixed effect model was used due to insignificant study heterogeneity (chi^2^ *p* > 0.05 and *I*^2^ <50%) (Figure 2 and Supplementary Figure 1). Meta-analysis was performed separately for reports on Asians and Europeans to identify significant differences in effect size, if any.

#### Allelic Association

*GC*1F allele has been found significantly predisposing for COPD outcome in combined analysis (Meta-OR = 1.29; 95% CI = 1.09–1.55; Z p-val = 0.004). Independently, *GC*1F allele has been found strongly associated among Asians (OR_Asia_ = 1.45; 95% CI = 1.24–1.68; Z p-val< 0.00001), but not among Europeans (OR_Europe_ = 1.02; 95% CI = 0.73–1.42; Z p-val = 0.92) (Figures 2A,B). Both *GC*1S and *GC*2 alleles were not found significant in conferring risk or protection with COPD outcome (Supplementary Figure 1). However, considering the trend of association, both these alleles were found protective in combined analyses.

#### Genotypic Association

Homozygous *GC*1F/1F was found significantly predisposing genotype with COPD outcome (Meta-OR = 1.61; 95% CI = 1.18–2.20; Z p-val = 0.002). Independent analysis found significant association of this genotype among Asians (OR_Asia_ = 1.93; 95% CI = 1.38–2.70; Z p-val = 0.0001), but it remains insignificant among Europeans (OR_Europe_ = 1.11 with 95% CI = 0.64–1.95; Z p-val = 0.71). Significant predisposition was observed for *GC*1S/1S genotype among Europeans (OR_Europe_ = 1.29; 95% CI = 1.00–1.68; Z p-val = 0.05), however it remains insignificant among Asians (Figure 2). Further, no significant associations were observed for any of the alleles or genotypes, either in combined or independent analyses in Asians and Europeans (Supplementary Figure 1).

### Sensitivity Analysis and Publication Bias

Sensitivity analysis was performed for each study. No significant deviation in heterogeneity and study significance (*p*-value of the χ^2^ and *Z*-test) was observed. Subgroup analyses did not identify any significant (p<0.05) subgroup stratification (Figure 2 and Supplementary Figures 1A–G). Further, manual investigation of funnel plots did not identify any publication bias, where shapes of the funnel plots were symmetrical (Supplementary Figure 2).

### Linkage Disequilibrium

Comparative LD analysis of GC locus showed substantial differences in the background LD structure between four reference populations. Comparatively similar LD structure was observed in CEU and GIH, however structure is further broken in JPT and YRI. Both the variations, rs4588 and rs7041, do not constitute any likely haplo-blocks in JPT and YRI (Supplementary Figure 3). Haplotypes for *GC*1F, *GC*1S, and *GC*2 were found to be present with relatively equal frequency among CEU (0.19, 0.57, and 0.24) and GIH (0.21, 0.46, and 0.32), however, these haplotypes were not found in JPT and YRI. Furthermore, moderate yet similar LD was observed between these two markers in CEU (*r*^2^ = 0.42; D' = 1) and GIH (*r*^2^ = 0.41; D' = 1), however, LD is completely broken in JPT (*r*^2^ = 0.10; D' = 1) and YRI (*r*^2^ = 0.00; D' = 0.53). Notable haplotypic variations were observed across the genomic region, whereas in JPT and YRI, these two variations are not in tight linkage with neighboring markers (Supplementary Figure 3). Allele frequencies of rs4588 and rs7041 and LD between them were seen to be very heterogeneous across 26 different populations, as documented in 1000 Genomes Project. Absolutely no LD (*r*^2^ = 0) was observed among different African populations, whereas the highest degree of LD was observed among Europeans and South Asian populations followed by Americans (Supplementary Table 2).

### *In silico* Functional Implications

*GC* (ENSG00000145321) expresses in the liver despite very negligible expression in the pancreas and stomach. For two missense SNPs, rs4588, and rs7041, no evidence was observed for significant eQTL on *GC* in liver tissue, however, significant eQTL was observed in subcutaneous adipose (*p* = 6.55E-6), sun exposed skin (*p* = 1.67E-6), and stomach (*p* = 5.46E-9) tissues. SNP rs4588 was identified to alter motif-binding sites of transcription factors SP1 and SP3; and transcription factor binding element (KLF16). rs4588 and rs7041 were both identified: (a) to localize in DNase hypersensitivity regions in a common set of cell types and tissues, and (b) potentially alter histone modification in liver (strongly) and skin (quiescent/low) tissue.

### Discussion

In this systematic review, we performed meta-analysis and evaluated linkage disequilibrium at GC locus, in order to investigate the association of common *GC* polymorphisms with COPD. This meta-analysis established that *GC*1F allele and *GC*1F/1F genotype confers risk of COPD. However, association is majorly restricted to Asians and not in Europeans. On the contrary, *GC*1S/1S genotype was observed to confer risk to Europeans only (with borderline significance). At least one copy of *GC2* has been found to confer protection from COPD among both Asians and Europeans. Previous meta-analysis studies and independent reports have shown different results from Europeans and Asians, which could be due to differences in allelic segregation and haplotypic heterogeneity at a population level (Chen et al., 2015; Horita et al., 2015; Wang et al., 2015; Xiao et al., 2015; Xie et al., 2015). Large differences in LD structure and haplotypes were observed in different ethnic populations, such as CEU GIH, JTP, and YRI. Although no reports are available (on association of *GC* variants and COPD) from African countries, we have included their representative genotypes for comparative genetic studies (Supplementary Figure 3). Notable differences in allele frequencies and LD between rs4588 and rs7041, among different populations, suggest significant population specific genetic contribution in *GC* variants (Supplementary Table 2). These major differences in LD between these two critical variants resulted into different haplotype frequencies and an absence of any quantifiable haplotypes in JPT and YRI. This indicates that perhaps different haplotypes are associated with different ethnic populations, which requires further large-scale genetic studies to uncover the novel alleles or haplotypes, if any. The overall trend shows relative similarity between CEU and GIH and distinct differences were observed in YRI and JPT. Different allelic arrangements of GC result into different GC variants, which vary in their isoelectric points and binding efficiency to vitamin D_3_ (Braun et al., 1992; Arnaud and Constans, 1993; Speeckaert et al., 2006). Furthermore, in different populations, rs4588 and rs7041 may tag different sets of regulatory and structural SNPs (in haplotypes) across *GC*, and thus could play critical role in regulating expression and function of the GC protein.

Although VDD is found to be associated with COPD (Jolliffe et al., 2018), the underlying causes for such mechanisms remain unanswered. Recent GWAS studies on COPD were unable to identify GC or vitamin D receptor (VDR) as a significantly associated gene (Wain et al., 2017). However, genetic polymorphisms from these genes are found to be associated with VDD (Yousefzadeh et al., 2014; Zaki et al., 2017). In most of the studies, low level of serum Vitamin-D_3_ is reported to be associated with the severity of COPD condition. Particularly, rs4588 has been shown to influence GC binding to Vitamin-D_3_ (Nimitphong et al., 2013). It can be argued that, along with sufficient vitamin-D_3_ intake/supplementation, a functionally more potent form of GC is necessary to maintain optimal serum bioavailability of vitamin-D_3_. Therefore, inter individual differences in the GC protein may act as a predisposing factor for COPD. Further genetic epidemiological studies are warranted to identify novel risk alleles from *GC* that are associated with *GC* function, and thus implication in COPD. However, the presence of differential LD structure of GC locus needs to be considered as a major confounding factor.

## Author Contributions

SS conceptualized and designed the study. RK and DN performed literature screening and meta-analysis. SS performed *in silico* genetic study. RK, DN, and SS contributed in writing the manuscript and interpreted the results. All the authors reviewed the manuscript and finalized for submission.

### Conflict of Interest Statement

The authors declare that the research was conducted in the absence of any commercial or financial relationships that could be construed as a potential conflict of interest.
