# Supplementary material for: Systematic Review and Meta-Analysis to Establish the Association of Common Genetic Variations in Vitamin D Binding Protein With Chronic Obstructive Pulmonary Disease
Source: Front Genet. 2019 May 16;10:413. doi: 10.3389/fgene.2019.00413 (PMC6532414; doi:10.3389/fgene.2019.00413)
Supplement: Supplementary file 1 [file Table_1.docx]

| **Study**  **Supplementary Table1 :** The demographic features, genotypic frequencies and allelic frequencies of subjects (cases and controls) included in the present study. | | **Cases/**  **Controls** | **Age** | | | | **Male/Female** | | | | **Smoking status(PY)** | | | | | **Genotypic frequencies** | | | | | | | | | | | | | | | | | | | | | | | **Allelic frequencies** | | | | | | | |
| --- | --- | --- | --- | --- | --- | --- | --- | --- | --- | --- | --- | --- | --- | --- | --- | --- | --- | --- | --- | --- | --- | --- | --- | --- | --- | --- | --- | --- | --- | --- | --- | --- | --- | --- | --- | --- | --- | --- | --- | --- | --- | --- | --- | --- | --- | --- |
|  |  |  |  |  |  |  |  |  |  |  |  |  |  |  |  | **1F-1F** | | **1F-1S** | | | | **1F-2** | | | | | | **1S-1S** | | | | **1S-2** | | | **2-2** | | | | **1F** | | | | **1S** | | **2** | |
|  |  |  | **Cases** | | **Controls** | | **Cases** | **Controls** | | | **Cases** | | **Controls** | | | **Cases** | **Controls** | **Cases** | **Controls** | | | **Cases** | | | **Controls** | | | **Cases** | | | **Controls** | **Cases** | **Controls** | | **Cases** | | **Controls** | | **Cases** | | **Controls** | | **Cases** | **Controls** | **Cases** | **Controls** |
| **Asian** | | | | | | | | | | | | | | | | | | | | | | | | | | | | | | | | | | | | | | | | | | | | | | |
| Ishii et al., 2001 | | 63/82 | 68.3 + 9.9 | | - | | 60/3 | 42/40 | | | 102.2 + 40.4 | | - | | | 23 | 17 | 15 | | 27 | | 16 | | 18 | | 1 | | | 5 | | | 6 | 8 | | 7 | | 2 | | 39 | | 40 | | 66 | 22 | 13 | 20 |
| Ito et al., 2004 | | 103/88 | 67.4 +7.8 | | 60.8 + 12 | | 99/4 | 72/16 | | | 58.3 + 29.1 | | 25.6 + 13.3 | | | 33 | 15 | 29 | | 27 | | 25 | | 30 | | 3 | | | 5 | | | 11 | 10 | | 2 | | 1 | | 120 | | 87 | | 46 | 47 | 40 | 42 |
| Lu et al., 2004 | | 69/52 | 61.1 + 7.4 | | 64.9 + 8.6 | | - | - | | | 820 + 429.7 | | 690.6 + 353.5 | | | 23 | 6 | 15 | | 16 | | 16 | | 14 | | 5 | | | 3 | | | 9 | 8 | | 1 | | 5 | | 77 | | 42 | | 34 | 30 | 27 | 32 |
| Huang et al., 2007 | | 75/69 | 60 + 6 | | 60 + 6 | | 75/0 | 69/0 | | | 37 + 4 | | 37 + 4 | | | 24 | 18 | 221 | | 18 | | 18 | | 20 | | 2 | | | 3 | | | 8 | 7 | | 2 | | 3 | | - | | - | | - | - | - | - |
| Shen et al., 2010 | | 100/100 | 62.3 + 9.7 | | 60.9 + 8.6 | | 72/28 | 66/34 | | | 123 + 29.7 | | 25.6 + 13.3 | | | 35 | 13 | 20 | | 26 | | 22 | | 29 | | 7 | | | 4 | | | 13 | 12 | | 3 | | 16 | | 56 | | 41 | | 24 | 23 | 10 | 22 |
| Maheshwari et al., 2014 | | 50/50 | 55.86 + 7.42 | | 55.82 + 7.89 | | 38/12 | 38/12 | | | 23.61 + 18.1 | | - | | | 6 | 2 | 10 | | 6 | | 8 | | 5 | | 8 | | | 15 | | | 15 | 10 | | 3 | | 12 | | 30 | | 15 | | 41 | 46 | 29 | 39 |
| Li et al., 2014 | | 116/134 | - | | - | | - | - | | |  | |  | | | 19 | 17 | 19 | | 32 | | - | | - | | - | | | - | | | - | - | | 9 | | 10 | | 94 | | 89 | | 61 | 105 | 77 | 74 |
| Jung et al., 2014 | | 203/157 | 67 | | 53 | | 194/4 | 148/9 | | | 46 +23.4 | | 30.7 + 17.4 | | | 45 | 34 | 41 | | 13 | | 7 | | 13 | | 56 | | | 33 | | | 32 | 34 | | 22 | | 12 | | 187 | | 132 | | 87 | 91 | 132 | 91 |
| Azzawi et al., 2017 | | 80/80 | 54.98 + 9.03 | | 53.875 + 7.742 | | 58/22 | 62/18 | | | 46.4 ­­+ 1.7 | | 33.7 + 1.2 | | | 12 | 6 | 23 | | 20 | | 9 | | 6 | | 19 | | | 18 | | | 16 | 13 | | 1 | | 7 | | 56 | | 38 | | 77 | 89 | 27 | 33 |
| **Caucasian** | | | | | | | | | | | | | | | | | | | | | | | | | | | | | | | | | | | | | | | | | | | | | | |
| Kueppers et al., 1977 | 109/109 | | 45-60 | | - | | - | | | - | | 30 | | 23 | | 62 | 57 | - | | | - | | - | | - | | - | | | - | | 46 | | 47 | | 1 | | 5 | | - | | - | - | - | - | - |
| Home et al., 1990 | 104/413 | | - | | - | | - | | | - | | - | | - | | 6 | 5 | 24 | | | 66 | | 3 | | 25 | | 40 | | | 141 | | 23 | | 134 | | 8 | | 42 | | 39 | | 101 | 127 | 482 | 42 | 243 |
| Laufs et al., 2004 | 102/183 | | 71.7 | | 42.9 | | 42/60 | | | 105/78 | | 38 | | - | | 1 | 2 | 11 | | | 24 | | 5 | | 8 | | 39 | | | 68 | | 35 | | 67 | | 11 | | 14 | | 9 | | 18 | 62 | 113 | 31 | 51 |
| Korytina et al., 2006 | 298/237 | | 61.52 + 12.7 | | 56.12 + 8.57 | | 238/60 | | | 153/84 | | 34.99 + 4.64 | | 32.45 + 3.5 | |  | | | | | | | | | | | | | | | | | | | | | | | | | | | | | | |
|  | Tatars  (131/106) | |  | | | | | | | | | | | | | 8 | 12 | 26 | | | 39 | | 25 | | 9 | | 30 | | | 14 | | 29 | | 25 | | 13 | | 7 | | 67 | | 72 | 115 | 92 | 80 | 48 |
|  | Russian  (166/130) | |  |  |  |  |  |  |  |  |  |  |  |  |  | 14 | 14 | 31 | | | 29 | | 20 | | 12 | | 42 | | | 25 | | 49 | | 45 | | 10 | | 5 | | 79 | | 69 | 164 | 124 | 89 | 67 |
| Janssens et al., 2010 | 253/150 | | 66(60-72) | 61(58-65) | | 215/49 | | | 120/32 | | | 47(33-63) | | | 39(30-52) | 8 | 4 | 35 | | | 29 | | - | | - | | - | | | - | | - | | - | | 16 | | 13 | | 87 | | 44 | 278 | 181 | 141 | 75 |
|  | | | | | | | | | | | | | | | | | | | | | | | | | | | | | | | | | | | | | | | | | | | | | | |
