# Supplementary material for: Systematic Review and Meta-Analysis to Establish the Association of Common Genetic Variations in Vitamin D Binding Protein With Chronic Obstructive Pulmonary Disease
Source: Front Genet. 2019 May 16;10:413. doi: 10.3389/fgene.2019.00413 (PMC6532414; doi:10.3389/fgene.2019.00413)
Supplement: Supplementary file 2 [file Table_2.docx]

| **Populations** | **Allele Frequency**  **rs4588-G/C** | **Allele Frequency**  **rs7041-A/T** | **LD (r2) between**  **rs4528-rs7041** |
| --- | --- | --- | --- |
| **Populations with African ancestry** | | | |
| ACB | -- | 0.85 | 0.00 |
| ASW | 0.95 | 0.83 | 0.00 |
| ESN | 0.95 | 0.92 | 0.00 |
| GWD | -- | 0.92 | 0.00 |
| LWK | 0.94 | 0.95 | 0.00 |
| MSL | -- | 0.95 | 0.00 |
| YRI | 0.97 | 0.91 | 0.00 |
| **Populations with American ancestry** | | | |
| CLM | -- | 0.41* | 0.35 |
| MXL | -- | 0.55 | 0.31 |
| PEL | 0.88 | 0.46* | 0.18 |
| PUR | 0.76 | 0.46* | 0.36 |
| **Populations with East Asian ancestry** | | | |
| CDX | -- | 0.64 | 0.20 |
| CHB | 0.70 | 0.73 | 0.15 |
| CHS | 0.68 | 0.69 | 0.21 |
| JPT | 0.78 | 0.76 | 0.10 |
| KHV | -- | 0.67 | 0.11 |
| **Populations with European ancestry** | | | |
| CEU | 0.71 | 0.43* | 0.42 |
| FIN | -- | 0.33* | 0.55 |
| GBR | -- | 0.43* | 0.47 |
| IBS | 0.73 | 0.44* | 0.48 |
| TSI | 0.74 | 0.45* | 0.40 |
| **Populations with South Asian ancestry** | | | |
| BEB | 0.74 | 0.47* | 0.41 |
| GIH | 0.68 | 0.54 | 0.41 |
| ITU | 0.70 | 0.45* | 0.53 |
| PJL | 0.70 | 0.45* | 0.52 |
| STU | 0.68 | 0.41* | 0.67 |

**Supplementary Table 2:** Comparative evaluation of allele frequency for rs4588-G/C and rs7051-A/T and linkage disequilibrium between them across different 1000 Genomes population. (Minor alleles are marked with *).
