# Supplementary material for: Systematic Review and Meta-Analysis to Establish the Association of Common Genetic Variations in Vitamin D Binding Protein With Chronic Obstructive Pulmonary Disease
Source: Front Genet. 2019 May 16;10:413. doi: 10.3389/fgene.2019.00413 (PMC6532414; doi:10.3389/fgene.2019.00413)
Supplement: Supplementary file 3 [file Data_Sheet_1.docx]

**Supplementary Figure 1: Results of meta-analysis showing risk imposed by GC alleles and genotypes.** Forest plots for alleles (a) *GC*1S, (b) *GC*2, and genotypes (c) *GC*1F/1S, (d) *GC*1F/2, (e) *GC*1S/1S, (f) *GC*1S/2, and (g) *GC*2/2.

**Supplementary Figure 2:** Funnel plots for GC1F, GC1S, GC2 alleles and GC1F/1F, GC1F/1S, GC1F/2, Gc1S/1S, GC1S/2 and GC2/2 genotypes.

**Supplementary Figure 3:** Linkage disequilibrium plots of CEU, GIH, JPT and YRI. (a) Overall LD background around 50kb window on both the sided around rs7041. The blue bar shows the exact location of rs4588 and rs7041, which corresponds with *GC*-1F, *GC*-1S and *GC*-2 alleles. (b) Different haplotypes reconstituted for this region.

**(a)**

**(b)**
